# General practitioner care of residential aged care facility residents at end of life: a systematic literature review and narrative synthesis

**DOI:** 10.1136/bmjopen-2025-104243

**Published:** 2025-11-12

**Authors:** Susannah Browne, Michael P Kelly, Ben Bowers, Isla Kuhn, Robbie Duschinsky, Charles Daniels, Stephen Barclay

**Affiliations:** 1 Public Health and Primary Care, University of Cambridge, Cambridge, UK; 2 Medical Library, School of Clinical Medicine, University of Cambridge, Cambridge, UK; 3 Palliative Care, Northwick Park Hospital, London, England, UK

**Keywords:** COVID-19, Delivery of Health Care, Integrated, Frail Elderly, Adult palliative care, Primary Health Care

## Abstract

**Abstract:**

**Objectives:**

In 2023, 21% of deaths occurred in residential aged care facilities (RACFs), a setting expected to play an increasing role in palliative and end-of-life care (PEoLC). General practitioners (GPs) oversee and deliver PEoLC in residential and nursing homes, yet little is known about their practice. We conducted a systematic review of the published evidence concerning how GPs provide this care: what they do and the quality, challenges and facilitators of that care.

**Design:**

Systematic review and narrative synthesis using the Preferred Reporting Items for Systematic Reviews and Meta-Analyses.

**Data sources:**

Medline, Embase, CINAHL, PsycINFO, Web of Science, Scopus and NHS Evidence and grey literature via Google Scholar were searched through 9 October 2024.

**Eligibility criteria:**

We included studies presenting new empirical data from qualitative, quantitative or mixed methods, were published in the English language and conducted in the UK, the European Union, Australia, New Zealand and Canada. We excluded studies with no new empirical data, discussion papers, conference abstracts, opinion pieces, study participants under 18 years old and in care settings other than RACF.

**Data extraction and synthesis:**

One independent reviewer used standardised methods to search and screen study titles for inclusion. This reviewer assessed all abstracts of the included papers, and a second independent reviewer screened 60% of the abstracts to validate inclusion. Risk of bias was assessed using Gough’s Weight of Evidence assessment. Thematic analysis was used to describe the contents of the included papers; a narrative synthesis approach was taken to report the findings at a more conceptual level.

**Results:**

The search identified 5936 titles: 35 papers were eligible and included in the synthesis. This is a nascent evidence base, lacking robust research designs and characterised by small sample sizes; the results describe the factors observed to be important in the delivery of care. Care provision is extremely variable; no models of optimal care have been put forward or tested. Challenges to care provision occur at every level of the care system. At macro level, service-level agreements and policies vary: at meso level, team-working, communication technology solutions and equipment availability vary: at micro level, GPs’ interests in providing PEoLC vary as does their training. No study addresses residents’ and relatives’ experiences and expectations of GPs' involvement in PEoLC in RACFs.

**Conclusions:**

The limited evidence base highlights that GP care at end of life for RACF residents varies greatly, with enablers and challenges at all levels in the existing care systems. Little research has examined GP PEoLC for RACF residents in its own right; insight is derived from studies that report on this issue as an adjunct to the main focus. With national policies focused on moving more PEoLC into community settings, these knowledge deficits require urgent attention.

**STRENGTHS AND LIMITATIONS OF THIS STUDY**

- This is a systematic review of the evidence base informing general practitioner (GP), palliative and end-of-life care of residential aged care facility residents.
- The database searches were thorough, created with help from a medical librarian coauthor; a three-component search strategy was adopted.
- Gough’s Weight of Evidence assessment was used to appraise the strength and quality of the evidence.
- Thematic analysis enabled data synthesis and an in-depth description of factors and issues concerning GP care provision.
- The literature is limited by methodological heterogeneity and small sample sizes: the voice of residents and their family members is absent from the literature.

## Introduction

 Residential aged care facilities (RACFs) are a major and growing location of palliative and end-of-life care (PEoLC) in more economically developed countries such as the UK^1^ and across the European Union.^2 3^ In 2022, 360 792 people were living in RACFs in England.^4^ In 2023, of the 581 363 deaths in England and Wales,^5^ 137 066 (20.9%) occurred in RACFs.^6^ Most people living in a RACF will die there.^7^ RACFs have been described as de facto hospices for many individuals over the age of 80,^8^ providing PEoLC akin to hospice care for the oldest old.^9^

RACFs are residential settings with onsite nursing staff (nursing home), social care staff only (residential home) or may provide both forms of care. We use the generic term residential aged care facility to refer to all of these; home care, that is, domiciliary care, is out of scope of this review. All RACFs are registered with and inspected by the Care Quality Commission.^10^ The great majority of RACF residents are older adults living with complex medical needs, multiple medical conditions and physical and/or cognitive frailty.^10^ More than 80% of UK RACFs are owned by private for-profit organisations and businesses: 63% of residents have care funded by the state and 37% of residents are self-funded.^11^

General practitioners (GPs) have a central role in clinically overseeing and providing PEoLC in the community, at home and in RACFs. The Royal College of General Practitioners describes GPs as the central point of contact for community PEoLC.^12 13^ Optimal PEoLC is considered to be a holistic approach,^14^ meeting people’s physical, psychological, social and spiritual needs^15^ and is recognised internationally as a human right to health.^16^ While there is a substantial literature concerning GP provision of community PEoLC,^17 18^ much less is known about GP PEoLC^19^ for RACF residents specifically.^20 21^ RACFs present a very different system of care to regular primary care, being situated within private sector care provision, social care policy and National Health Service (NHS) care.

In England and Wales, primary care networks (PCNs) designate a lead GP for each RACF in their area, as specified by the ‘Enhanced Health in Care Homes’ framework^10^ and the ‘Network Contract Enhanced Direct Service’ agreement.^22^ This guidance stipulates, but is not limited to, regular (weekly) GP-led RACF ‘ward rounds’, coordination of the PCN multidisciplinary team (MDT), prioritisation of residents for clinical review, ensuring that a treatment plan is in place (in consultation with residents and relatives) within 7 days of admission and a holistic assessment of PEoLC needs if appropriate.

Previous reviews of PEoLC for RACF residents have highlighted its complexity and multifaceted nature^23 24^ as an interplay of numerous, structural, clinical and interpersonal factors.^25^ While GPs are central in providing community PEoLC,^10^ they face many challenges in doing so, including time pressures,^25 26^ fragmented MDT working,^25 26^ interdisciplinary communication issues, lack of continuity of care, clinical and symptom management issues,^27^ lack of training in PEoLC,^28^ difficulties with anticipatory prescribing,^29 30^ variable interest in and engagement with PEoLC^31^ and difficulties accessing specialist PEoLC support and services.^32^ The impact of these multifaceted challenges in the RACF context is unknown.

The COVID-19 pandemic put RACFs, primary care and palliative care services into severe difficulties in the UK.^33^ The emerging studies of the impact of the pandemic on PEoLC in RACFs have mainly considered the role of advance care planning (ACP),^34 35^ with others reporting the breakdown of relationship-centred care^36^ and the contrasting impact between PEoLC community and acute settings.^37^ The specific impact on GP provision of PEoLC for RACF residents has not been reported to date.

RACF healthcare is unique in that it spans the private sector, health and social care systems and will be of increasing importance in the future as the population ages and policies shift.^38^ With increasing pressures on GP and primary care teams, understanding how GPs provide RACF PEoLC is a priority knowledge need in order to determine the optimal structure, role and processes to support GPs in delivering this care.

### Aims

To review the published evidence about GP PEoLC provision for RACF residents.

### Review questions

With regard to GP care of RACF residents at the end of life:

1. In what ways do GPs provide care?
2. What is the quality of GP care?
3. What constitutes good GP care and what are the enablers?
4. What works less well in GP care and what are the challenges?
5. What was the impact of the COVID-19 pandemic?

## Methods

### Data sources

Searches of the peer-reviewed literature in three target domains (GP, RACFs, PEoLC) were undertaken in seven databases (Medline and Embase via OVID, CINAHL and PsycINFO via EBSCOhost, Web of Science Core Collection, Scopus and NHS Evidence) and searched from inception by a medical librarian (IK). An initial scoping search identified no publications that addressed all three domains: the search strategy was revised to include physician, family physician, family doctor; residential care, nursing home and long-term care; palliative care, dying and terminal care. ‘RACF’ was defined as any residential setting with or without onsite nursing provision, which is not a resident’s own domestic dwelling. ‘GP’ was defined as a doctor working in the community who had responsibility for the care of RACF residents.

The final search strategy for Medline via OVID is shown in box 1. The full search strategies are provided in online supplemental material 1.

Box 1 **Search strategy for Medline via OVID**

1. one residential facilities/ or group homes/ or homes for the aged/ or exp nursing homes/ or institutionalizationinstitutionalisation/ or Long-Term Care/ or Housing for the Elderly/ or (((care or nursing or residential or rest or old* people* or old folk* or group or geriatric or elderly) adj2 (home or homes)) or ((long term or long-term or residential or institution*) adj care) or ((aged or elderly or geriatric or extended) adj2 care adj2 (facility or facilities)) or ((aged or elderly) adj3 (home or homes))).mp. 158 267
2. two exp Terminal Care/ or exp Palliative Care/ or exp “Hospice and Palliative Care Nursing”/ or exp death/ or exp Palliative Medicine/ or exp Terminally Ill/ or ((end adj2 life) or ((final* or last*) adj1 (hour* or day* or minute* or week* or month* or moment*)) or palliat* or terminal* or (end adj stage) or dying or (body adj2 (shutdown or shut* down or deteriorat*)) or deathbed).mp. 880 717
3. three exp General Practitioners/ or exp physicians, family/ or (gp or general practi* or ((family or primary care or primary healthcare) adj3 (doctor* or physician*))).mp. 173 382

The initial search, undertaken in March 2021, was updated in March 2022, October 2023 and October 2024. Table 1 shows the number of papers identified by each database.

**Table 1:** Search results by database

	Original March 2021	Additional hits February 2022	Additional hits October 2023	Additional hits October 2024
Medline	704	76	83	45
Embase	840	75	145	43
CINAHL	482	31	25	21
PsycINFO	229	5	12	4
Scopus	1305	86	134	130
Web of Science Core Collection	1020	103	146	79
NHS Evidence	108	5	n/a	n/a
Total	4688	381	545	322
Total deduplicated	2779	221	352	192

Searches run 12 March 2021.

Search rerun 15 February 2022.

Searches rerun 16 October 2023.

Searches rerun 9 October 2024.

NHS Evidence was withdrawn as a resource by the National Institute for Health and Care Excellence in March 2022, so was unavailable to use to update the search in October 2023.

A grey literature search was conducted in January 2022 and February and October 2024 using the Google Search terms in box 2.

Box 2 **Grey literature search strategy**

“nursing home” “end of life” filetype:pdf

“home for the aged” “end of life” filetype:pdf

“residential home” “end of life” filetype:pdf

“old folks home” “end of life” filetype:pdf

“nursing home” “general practitioner " filetype:pdf

“home for the aged” “general practitioner " filetype:pdf

“residential home” “general practitioner " filetype:pdf

“old folks home” “general practitioner” filetype:pdf

“nursing home” “family doctor” filetype:pdf

“home for the aged” “family doctor” filetype:pdf

“residential home” “family doctor” filetype:pdf

“old folks home” “family doctor” filetype:pdf

“end of life” “general practitioner” filetype:pdf

“end of life” “family doctor” filetype:pdf

Reference and citation searches of included papers yielded seven additional papers. A PhD thesis^39^ reference search yielded no papers.

### Inclusion and exclusion criteria

Inclusion and exclusion criteria were developed using the PICOTS framework (Population, Intervention, Comparator/Context, Outcome, Timing and Setting).^40^ Studies were included if they presented new empirical data from qualitative, quantitative or mixed-methods studies, were published in the English language and conducted in the UK, the European Union, Australia, New Zealand and Canada (countries with similar systems of GP provision). Exclusion criteria included studies with no new empirical data, discussion papers, conference abstracts, opinion pieces, study participants under 18 years old and care setting other than RACF (eg, hospital, home, prison, hospice, specialist care settings for people with learning or other disabilities).

**Figure 1:**
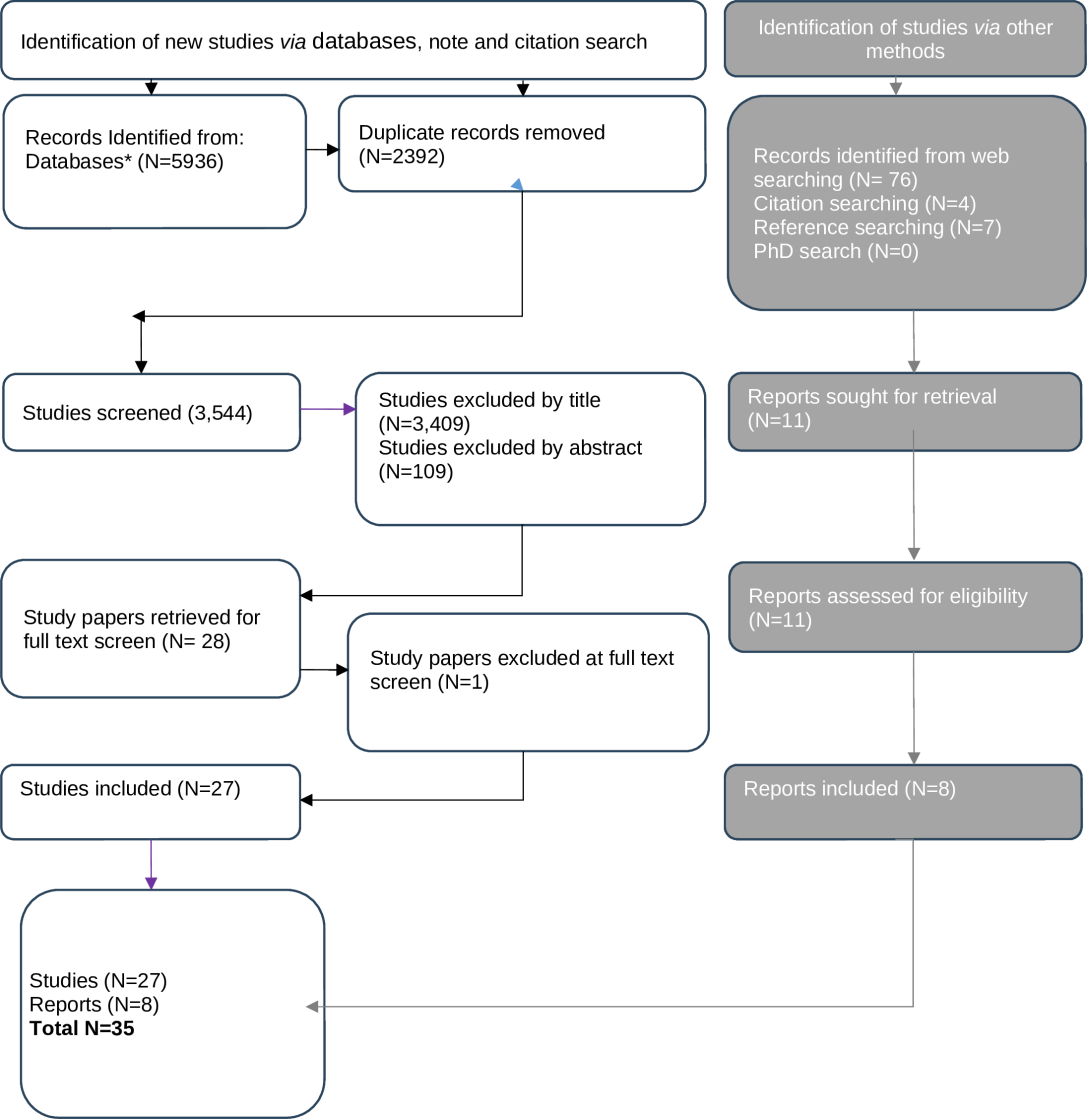
Preferred Reporting Items for Systematic Reviews and Meta-Analyses diagram.

Search results were de-duplicated and uploaded into EndNote (V.x9). The Preferred Reporting Items for Systematic Reviews and Meta-Analyses flow diagram is shown in figure 1.^41^,^41^

### Study selection and data extraction

SBr conducted the full title and abstract screening. BB independently screened 16 abstracts (62%) to validate SBr’s approach. There were no disagreements. SBr assessed all full texts (n=35) and undertook data extraction into a review-specific data extraction sheet (online supplemental material 2), conferring with BB and SBa where there was uncertainty. EndNote (V.X9) reference management software was used to sort through and record the papers as they were included or excluded at each stage of the search, title, abstract and full-text read.

### Quality appraisal

Gough’s Weight of Evidence (WoE) framework was used to assess the quality and relevance of included publications.^42^ This assesses the quality and applicability of the evidence for the review questions across three domains:

A=internal validity of study.
B=appropriateness of study design to review aims.
C=focus or relevance of study to review aims.

Three scores (high, medium, low) for A, B and C are then combined to provide an overall WoE score D. WoE scores were calculated by SBr for all included papers. SBa and BB each independently scored 50% of the included studies with WoE scores compared, and consensus was achieved.

### Data synthesis

A data extraction sheet was used to record the findings of included papers against the review questions. These data were then uploaded into NVivo 12, which facilitated the identification of content and themes against each review question. Line-by-line coding was used to sort the data into semantic themes by research question. These were then conceptualised into macro, meso and micro levels of the system of care to aid reporting and reflection on the findings. An inductive narrative synthesis of the extracted data was conducted,^43 44^ allowing the findings of the heterogeneous methods and aims and qualitative and quantitative findings to be included and reported in an integrated way.^45 46^ A three-stage iterative analysis was performed:

1. Each study was tabulated according to study research questions, methods, population, study results and the review questions the data reported against.
2. SBr then conducted a thematic analysis to identify the emergent themes that addressed each review question.
3. The WoE scores for each paper and the degree to which the data informed the synthesis were considered.

All data were included in the synthesis, regardless of WoE score, as only seven papers directly addressed all the search domains and study sample sizes were commonly small. WoE scores can be viewed in online supplemental material 4.

### Patient and public involvement

Patient and public involvement consultations were held with three lay advisors with personal experience of end-of-life care for family members, and a range of PEoLC healthcare professionals through research workshops and consultation events. Both the lay advisors and the PEoLC professionals agreed that the subject of the review was a gap in current knowledge and that this review was a timely piece of work.

## Results

A range of research methods were employed in the studies identified: mixed methods (n=10), qualitative including focus groups, interviews and ethnography (n=12), quantitative including surveys and clinical record reviews (n=12) and Delphi technique (n=1). Due to the methodological limitations and small sample sizes of the included studies, we adopted a descriptive approach to reporting the studies. It was not possible to undertake meta-analysis or meta-synthesis. The methodological details of the included papers and overall WoE scores were 10=high, 16=medium and 7=low and can be found in online supplemental material 3.

The 35 included studies were from the UK (n=10), Australia (n=7), PACE study (Belgium, the Netherlands, England, Finland, Poland, Italy and Switzerland) (n=4), Germany (n=4), Canada (n=2), New Zealand (n=2), Belgium (n=1), the European Union (n=1), Denmark (n=1), Norway (n=1), Sweden (n=1) and the Netherlands (n=1).

The three search domains (GP, PEoLC, RACFs) were directly addressed by 9 papers: in the other 26 papers, they were addressed as a subset or adjunct to the studies’ main research questions. For ease of reporting, the disparate factors reported to constitute good or poor care (questions 3 and 4) are structured into macro (governance and policy), meso (local/group) and micro (individual) factors.

Table 2 shows the review questions addressed by the included papers.

**Table 2:** Review questions addressed by the included papers

Paper number	Author	Q1	Q2	Q3	Q4	Q5
1	Allers *et al*^65^		•	•	•	
2	Andrews *et al*^52^	•	•	•	•	
3	Badger *et al*^51^	•	•	•	•	
4	Balmer *et al* 2020^66^		•		•	
5	Banerjee *et al* 2018^21^	•		•	•	
6	Baranska *et al* 2020^62^	•	•			
7	Borbasi *et al* 2021^61^	•		•	•	
8	Bauer *et al* 2024^75^		•			
9	Ding *et al* 2022^60^	•			•	
10	Dreyer *et al* 2011^48^	•	•	•	•	
11	Dujardin *et al* 2021^77^					•
12	Forbat *et al* 2024^76^		•			•
13	Frey *et al* 2020^74^				•	
14	Froggatt *et al* 2009^53^	•			•	
15	Gorlen *et al* 2013^69^			•	•	
16	Grune *et al* 2021^73^				•	
17	Handley *et al* 2014^47^	•		•	•	
18	Harasym *et al* 2020^72^			•	•	
19	Harasym *et al* 2021^71^					
20	Kinley *et al* 2014^49^		•			
21	Kiresbom *et al* 2017^54^	•		•	•	
22	Mitchell *et al* 2022^27^	•				
23	Nilsen *et al* 2024^1^	•		•	•	•
24	Ong *et al* 2010^70^		•	•	•	
25	Oosterveld-Vlug *et al* 2018^50^	•	•			
26	Phillips *et al* 2009^67^		•	•	•	
27	Pulst *et al* 2021^56^	•				
28	Rainsford *et al* 2020^57^	•		•	•	
29	Rainsford *et al* 2022^68^			•	•	•
30	Sidell *et al* 1997^58^	•		•	•	
31	Seymour *et al* 2011^64^		•	•	•	
32	Ten Koppel *et al* 2019^63^		•			
33	Tuckett *et al* 2013^55^	•			•	
34	Tuckett *et al* 2015^55^			•	•	
35	Vandervoort *et al* 2014^59^	•	•			

GP, general practitioner; Q1, In what way do GPs provide care?; Q2, What is the quality of GP care?; Q3, What constitutes good GP care and what are the enablers?; Q4, What works less well in GP care and what are the challenges?; Q5, What was the impact of the COVID-19 pandemic?.

As table 2 shows, four papers on the impact of COVID-19 on GP PEoLC for RACF had been published by the end of October 2024. These looked at ACP, telemedicine and symptom control in the care home context during the pandemic. A challenge to GP care was the research question that was most discussed in the research papers.

Online supplemental material 4 shows the themes that each paper responded to. The most commonly reported issues with GP PEoLC were

- Personal factors of the GP, that is, did they want to and were they skilled to care.
- The challenges presented for both the GP and families of negotiating care of the dying resident between them.
- The consistency of GP care, that is, the same GP managing care was helpful; different GPs meant care could deviate from the ACP.
- Communication across all parts of the system, for example, access to notes, updates to ACPs being accessible, etc.
- Problems with symptom control, GPs not knowing doses of anticipatory medication, for example, or not knowing about palliative and end-of-life care.

The following sections examine in detail the content of the included papers according to the research questions of this review. As there is such a volume of factors to present that facilitate and challenge GPs providing care in the RACF context, these are structured into macro, meso and micro factors, that is, at governance level, local delivery level and individual/personal factors.

### Review question 1: In what way do GPs provide care for RACF residents at end of life?

GPs were the most widely accessed healthcare service for RACF residents^47^ and described as being formally responsible for residents’ medical care.^48^ One study reported that 96% of residents had a GP visit in the last 6 months of life: 50% had an out-of-hours (OoH) GP visit in the last year of life and 37% in the last month.^49^ GP visits increase in the last days of a resident’s life.^47^ UK residents received fewer GP visits in the last 3 months of life compared with Italy, the Netherlands, Belgium, Poland and Finland.^50^

While GP RACF visits are seen as central to care provision,^51^ these often have no set pattern, being either regular weekly rounds or ad hoc,^52^ dependent on contractual and financial arrangements and residents’ healthcare needs.^53^ GP visits were highly valued by residents, RACF staff and other visiting professionals, and prioritised over other activities; relatives and visiting professionals arranged to be present when the GP visited (generally after morning or afternoon surgery).^52^

GP general care of residents includes general medical management^4748 53^,^55^; arranging investigations^54^; prescribing medicines^54^; treatment decision-making^4756^,^58^; medication reviews^21^ and decisions concerning hospital admissions.^21 56 57^

GPs’ PEoLC-related tasks include drawing up advanced care plans^27 54 57 59^; palliative care and symptom management^54 55 57^; anticipatory medication prescribing^55 57^; care coordination^27 55 60^; RACF staff and relative education^21^; facilitating acceptance of palliative care and advocating for a palliative care approach, especially for residents with dementia.^61^

GP communication about residents’ clinical state, mental state and management options is central to clarifying families, health and care professionals’ expectations of care.^55^ This involves communication with relatives,^5557 61^,^63^ RACF staff^60^ and other health and social care professionals.^21 60^ Communication is via health and other care records,^48 54 55^ in person and via telephone or video.^60^

### Review question 2: What is the quality of GP care for RACF residents at end of life?

Quality of care was not a primary research question but PEoLC-specific markers of good GP resident care were reported to be supporting RACF residents and staff,^64^ residents having ACPs,^65^ recognition of residents’ terminal phase^50^ and involvement of specialist services.^49^

Signifiers of more general good GP resident care include GP availability^48 66^; contracted GP time for visits^48^; coordinated care^66^; involving other healthcare professionals^48^; good communication^48 62^; ensuring consensus about goals of care^48^; use of frameworks for care^48^; consistency of medical records^48^; good GP and RACF staff working relationships^66^; and proactive strategies to manage the RACF workload (eg, allocating enough time to support care needs).^67^ Regular weekly GP ‘ward’ rounds are valued by residents, relatives and nursing home staff.^52^

Care was less good if families intervened in the care plan by wanting active treatment^66^; when there was inadequate pain control^58^ and care was shared between multiple GPs.^48^

### Review question 3: What constitutes good GP care for RACF residents at end of life and what are the enablers?

#### Micro/individual GP attributes

GPs are reported as providing good care if they show skill, experience and confidence in PEoLC^61 68^; thoroughness^54^; reliability^69^; ability to recognise the end-of-life phase^67^; ability to make decisions in the face of uncertainty, complexity and multimorbidity^21 54 58^; and an integrated perspective of quality of life.^67^

The following foci of care were considered essential: prioritising comfort and dignity^67^ and high-quality end-of-life care^67 70^; planning care ahead of time, including OoH^64 69^; spending time with residents to understand their care preferences; communicating early about PEoLC and managing expectations of treatment.^21^

GP activities viewed as components of good care included regular GP RACF rounds^52^; regular medication reviews^54 67^; managing pain well^58^; ensuring that comprehensive ACPs are in place,^54^ which include residents’ wishes beyond ‘no resuscitation’ orders^57^ and ‘do not transfer to hospital’ orders^54 67^; and flexibility in actioning ACPs according to the current context.^57^

#### Meso/local or group factors

Good care is enabled by personal continuity of the visiting GP^52^; working well with others, including other healthcare professionals, RACF staff, residents and their relatives^51^; developing trusting working relationships^54 58 61 67 68 71^; being seen as an ally by the RACF staff^61 68^; providing mentorship^71^; being open and responsive^61^; involvement of residents and relatives^21 61 67^; making time for conversations with relatives^52^ and involving them in ACP conversations^52^; and advocating for and facilitating the acceptance of palliative care.^61^

GP RACF ‘ward rounds’ are viewed as key enablers of inclusivity and working well with others,^52^ as are involvement of other services including for investigations, involvement of specialists including old-age psychiatrists and neurologists^21^ and palliative care specialists.^47 71^

#### Macro, structural and governance factors

Governance conditions, including a contract that stipulates GP visits, enable good care through consistency, accountability and provide a framework of authority.^51 58^

### Review question 4: What works less well in GP care of RACF residents at the end of life and what are the challenges?

A very large amount of data addressed this question. Challenges to good care were reported at macro (governance and resourcing), meso (local policy and working relationships) and micro (personal attributes and skills) levels.

#### Macro and structural factors

These barriers to good care include financial, including under-resourcing of the RACF sector^54 58^; human resources, including shortages of GPs^67^ and social care and nursing staff^54 68^; weak frameworks for guidance of GP-RACF staff collaboration^48^ and complexity of social care governance.^67^

#### Local/meso factors

At the local or meso level, multiple factors were outside of GP control, which had a negative impact on their care. These included availability of medical equipment^66^; RACF environment, including shared bedrooms^67^; and insufficient access to palliative care, geriatrician and mental health specialists.^65 72^

##### Workforce factors

Workforce challenges include recruitment and retention^47 52 58^; poor quality of care provision,^65^ lack of confidence in staff abilities,^51 54 57 72^ including ability to recognise symptoms of distress^54^; insufficient staff training^66^ and status issues hindering collaboration.^51 57^

The OoH period (nights and weekends) presents major challenges.^66^ Poor quality care by OoH services^64 70^ includes OoH clinicians not being familiar with existing care plans^54^ and being reluctant to make major management decisions for unfamiliar patients,^51^ including hesitation to prescribe anticipatory medication.^51^ These factors may contribute to potentially avoidable hospital admissions OoH.^21 70^

##### GP engagement

Variable GP engagement with RACF resident care in general was a common theme,^64^ influenced by GP time pressures, remuneration and lack of clear frameworks for resident care and visits.^47 48 51 55 67^ At times, GPs are unavailable to make key decisions or attend to emergencies due to other patient commitments.^54 56 57^ RACFs lack clear PEoLC policies, leaving staff unclear about when to contact GPs.^47^ Multiple GPs being responsible for a RACF impacted on continuity of care.^48 51 53^

##### Advance care planning

ACP conversations at times do not happen^21 47 70^ or may not include residents.^59 70^ GPs may not have the full picture of the patient,^55^ leading to ACPs being out of date,^54^ not covering acute situations^48 64^ or containing ambiguous goals of care.^57^ Factors leading to ACPs not being in place include GP workload, uncertainty over which residents need assessment, changes in residents’ health status and time constraints limiting conversations with residents and their families^54^; uncertainty whether GP or RACF staff should have conversations^47^ and resident illness preventing conversations happening.^47^

There is concern that ACP conversations add a burden to an already overextended workforce.^72^

While approximately 40% of residents in one study had an ACP, only 30% of these contained instruction on hospitalisation, and in 40% of those the instructions were ignored.^65^ Having an ACP was not positively correlated with the quality of dying.^59^

##### Coordination of care and continuity of care

Difficulties with coordination of care between health and social care systems and professionals include difficulties in coordinating care with RACF staff^52 67^; lack of discussions between the GP, other professionals, residents and relatives^52^; poor coordination between hospital doctors, RACFs and GPs at discharge to RACF.^48^

##### Communication

Poor inter-professional communication challenges include^57 66^ inadequate flow of information between GPs and RACF staff^73^; limited GP time at meetings or during visits^73^; GP referrals to specialist services^58 70^ and arranging equipment, including syringe drivers.^64^

Communication via residents’ records is limited by lack of staff IT skills,^48^ incompatible IT systems between the RACFs, GPs and hospitals,^21^ which at times leads to GPs having to print, scan or deliver hospital discharge and drug charts notes by hand.^54^

Communication with relatives is a major factor^54 62 70 74^; GPs may not see liaising with relatives as a part of resident PEoLC.^58^ Cultural and language barriers may lead GPs to leave communication with residents and relatives to RACF staff.^47 48 55^

##### Resistance from others

At times, other individuals impact GP care. Staff or relatives may pressure the GP to prescribe medication^21 71 73^ or try to influence medication administration and clinical management decisions.^66 72^

### Micro/personal factors

#### Personal GP attributes

Individual GP factors that influence care provision include reluctance to visit RACFs^69^ or to regularly review residents^51 70^; regarding PEoLC as burdensome^21 48 51 58 61 67^; being reluctant to prescribe anticipatory medication^51 64 72^ or prescribing without seeing the resident.^52^ Inexperienced GPs may find PEoLC particularly challenging.^54^ No GPs reported involvement in bereavement care of families or RACF staff after a resident’s death.^58^

#### GP training and experience

GPs may have had limited palliative care training and experience,^48 51 58 61^ which may lead to limited symptom assessment and management,^58 67 72^ lack of understanding of anticipatory prescribing and syringe driver use,^57 58^ limited understanding of services^58^ and difficulty recognising residents nearing death.^47^ GP training in PEoLC is variable across countries.^75^

#### Communicating with relatives and families

GPs recognise the importance of reaching consensus with relatives about residents’ care, although some only communicate with relatives if there are problems.^57 67 72^ This can be challenging as relatives may not see the resident as approaching the end of life, may have unrealistic expectations of treatment options^58^ or think GPs are withholding treatment.^55 72^ At times, families may challenge GPs and seek to impose their rights as decision-makers in care planning^5560^,^72^ and become unduly emotional or adversarial which GPs report as challenging.^55^ This can have profound impacts on residents’ care by GPs either continuing life-prolonging treatment or restricting treatment^48^; as a last resort in managing conflict with families, GPs admit residents to hospital or withdraw from the resident’s care completely.^73^

In turn, relatives reported communication with GPs to be a challenge^74^ and saw poor GP communication as signalling a lack of interest and respect.^60 74^

#### Resident characteristics

Consultation with residents was rarely mentioned, beyond GPs being less likely to discuss PEoLC with RACF residents than with patients at home.^60^

At times, resident characteristics present a challenge for GPs. They can be too frail to transport to hospital for investigation or treatment, leaving GPs working with unknowns,^21^ and there may be no discrete identifiable trigger to indicate a frail elderly patient as being at the end of life.^47 54 57^

During the initial assessment on becoming resident at the home, time restrictions, limited contact with and the apparent wellness of residents inhibited conversation about PEoLC.^76^ Discussions of deterioration or ACPs were not found in residents' notes.^76^ GPs may assume that dying older people were aware of what was happening, were accepting of death and happy to leave their care in the hands of the GP.^58^

### Research question 5: How has GP end-of-life care for RACF residents changed during and following the COVID-19 pandemic?

To date, four papers have addressed this question. The pandemic depleted GP support to RACFs, increasing the pressure on RACF staff.^76^ COVID-19 provoked GPs to more thoroughly engage with residents’ relatives^1^ and become more central in devising ACPs^77^ by discussing treatment options and putting structured approaches to care in place to support residents who were dying.^1^ In some cases, GPs used PEoLC medication at higher doses than customary, which was successful, but placed an additional burden on RACF nurses.^1^ The move from in-person to digital consultations during the pandemic was problematic as non-verbal cues and relatives were absent from consultations.^68^

## Discussion

This is the first systematic review that describes what GPs do in providing PEoLC for RACF residents, how well they do it, what constitutes and enables good care, the challenges to that care (the majority of the findings), how care may become less than optimal and the impact of the COVID-19 pandemic on GP care of care home residents at end of life. GPs are at the centre of PEoLC for residents; we highlight the multifactorial dependencies that intervene in its practice. The most reported factors as either facilitating or challenging GPs' PEoLC for RACF residents were individual GP factors; working with residents’ families; consistency of GP providing the caring; communication and symptom management.

The majority of RACF residents will require GP care at the end of their lives. However, there is a large amount of variability in the care they receive. GP care is highly valued by RACF staff for its skilled medical expertise and for its leadership role in coordinating the input of other health and care professionals.

The findings of this systematic review are consistent with those reported by previous systematic reviews examining GP PEoLC but extend them beyond a focus on general healthcare in RACF^20^ and community PEoLC^17 18^ into the RACF-specific PEoLC space. Previous studies which have considered RAC PEoLC and GP involvement have examined factors such as recognition of residents’ terminal phase,^50^ communication skills,^62^ interventions to improve care ^55 68^ and GPs’ views.^65^,^67^ This review confirms the findings of these studies and moves beyond them to highlight the multitude of factors that can intervene in the delivery by GPs of PEoLC for RACF residents.

GPs’ role in RAC PEoLC as reflected by the findings of this review blends clinical expertise, leadership, teamwork, management and operational delivery factors, some of which are outside of their control. The factors which determine GP PEoLC for RACF residents are a combination of micro, meso and macro issues: personal GP factors, including attitude, experience and training; resource factors, including time to spend with residents, continuity of care, ACP, resources and funding, and interpersonal working relationships through communication and record keeping; and policies and frameworks which stipulate and resource when and how GPs care for RACF residents at end of life.

When PEoLC of RACF residents is working well, the GP is reported to be confident, skilled and forward thinking, one who uses a structured and documentary approach and is prepared to be flexible in the face of dynamic symptoms; there is continuity of care in which the same GP attends the resident and is supported by a clear service-level agreement which stipulates and funds weekly ward rounds.

In the absence of the above, there are multiple layers at which the system of GP care of RACF residents can prove challenging, from the lack of resources and governance agreements to equipment availability, skillsets and willingness. All, singular or combined, can have a negative impact on the resident’s final days of life.

During the COVID-19 pandemic, GPs restricted in-person visits to RACFs, which placed an additional burden on RACF staff. The pandemic highlighted the need for ACP to be in place and of involving relatives in planning discussions. Digital consultations replaced in-person contact, thereby losing the nuance brought by non-verbal/contextual issues in appraising health status. GPs developed innovative approaches to symptom management using increased levels of PEoLC medications to manage the symptoms of COVID-19 at end of life.

There are notable gaps in the literature. For example, the quality of GP care has not been specifically addressed. Some factors which reflect good GP care can be inferred from the literature. From a medical perspective, these include ACPs, recognition of the end of life phase and weekly ‘ward rounds’. Some broader skills too are markers of good care such as working well with others and supporting colleagues. Poor pain control infers poor PEoLC.

The views of RACF residents are unaccounted for in the literature. What do they expect from their GP when considering their end-of-life care and why are they absent from the literature? Similarly, the views of relatives are absent. When relatives are mentioned in the literature, they are positioned as challenging the GP and being difficult to work with, but what are their needs? In addition, despite holistic care being regarded as optimal PEoLC, the GP’s role in psychosocial and spiritual care is unaddressed, as is their role in bereavement care for family and RACF staff when a resident dies. All three of these issues are fundamental in descriptions in the literature of what optimal PEoLC should contain but remain absent from the literature examining GP care of RACF residents at end of life.

GP RAC PEoLC care is delivered within a complex adaptive system at the interface of healthcare, social care and private enterprise. Although GPs are responsible for the PEoLC of RACF residents, their level of control in this context is variable and not considered in the literature. For example, how does a GP interested in improving RACF PEoLC influence a private sector provider to implement structural changes to improve care or increase resources for PEoLC? Outcome measures or measures of quality of care are not stated in the papers, and untested assumptions about the markers or drivers of good care such as ACPs are made. Nevertheless, it can be inferred from the published research reported here that good GP PEoLC for RACF residents is associated with reduced hospitalisations, appropriate symptom control and satisfaction reported by those receiving care, their relatives and other health and social care professionals involved in caring.

With renewed policy commitment to move more palliative and end-of-life care from hospitals into community settings,^78^ it is imperative that the evidence base for GP care of RACF residents at end of life is developed. Specifically, more research should focus on determining what marks good GP PEoLC for RACF residents, how can the barriers to good care be overcome in this setting and how good GP care across RACFs be scaled up to ensure equality of access.

## Conclusion

This systematic review of the literature highlights the limited research concerning the role of GPs in PEoLC for RACF residents: the voice of residents and relatives is absent from the literature as is reference to GPs’ role in holistic and bereavement care. From the evidence gathered here, GP input at the end of life varies widely, which means that the quality of RACF resident death is extremely variable too. Good GP care combines clinical skills and relational multidisciplinary teamworking and is supported by adequate information management, resource and governance systems: the quality-of-care provision is variable. COVID-19 disrupted the system and highlighted both strengths and weaknesses of GP RACF PEoLC. The value of ACP was highlighted, and novel approaches to PEoLC medication proved fruitful in providing comfort. General practice now has a strategic opportunity to take these lessons and evidence forward and develop new and better ways of working.

## Implications for research and practice

PEoLC in RACFs is a specific context given its location on the interface of social care, primary healthcare and private enterprise. There is a pressing need for

- Policy to clearly define and resource the roles and expectations of GPs in RACF PEoLC, with explicit guidance, service-level agreements, adequate funding and measurable outcomes.
- Research to identify residents’ and relatives’ expectations of GP care and the implications for practice.
- GPs to work with colleagues across the health and social care system to ensure that optimal and truly ‘patient-centred’ care is provided for RACF residents at end of life.

## Supplementary material

10.1136/bmjopen-2025-104243 online supplemental file 1

10.1136/bmjopen-2025-104243 online supplemental file 2

10.1136/bmjopen-2025-104243 online supplemental file 3

10.1136/bmjopen-2025-104243 online supplemental file 4

## Data Availability

All data relevant to the study are included in the article or uploaded as supplementary information.

## References

[1] Nilsen A, Eriksen S, Lichtwarck B (2024). Treatment and care for nursing home residents with COVID-19: a qualitative study. J Multidiscip Healthc.

[2] Murtagh FEM, Bausewein C, Verne J (2014). How many people need palliative care? A study developing and comparing methods for population-based estimates. Palliat Med.

[3] Van den Block L, Smets T, van Dop N (2016). Comparing Palliative Care in Care Homes Across Europe (PACE): protocol of a cross-sectional study of deceased residents in 6 EU countries. J Am Med Dir Assoc.

[4] Statistica (2022). Statista. https://www.statista.com/statistics/1082379/number-of-people-living-in-care-homes-in-the-united-kingdom/.

[5] ONS. Office for National Statistics (2023). ONS - deaths registered in england and wales: 2023. https://content.govdelivery.com/accounts/UKONS/bulletins/3bb2187.

[6] ONS (2023). Deaths of racf residents, england and wales - office for national statistics. https://www.ons.gov.uk/peoplepopulationandcommunity/birthsdeathsandmarriages/deaths/bulletin%20s/deathsinthecaresectorenglandandwales/2022.

[7] ONS RACF resident deaths registered in england and wales, provisional - office for national statistics. https://www.ons.gov.uk/peoplepopulationandcommunity/birthsdeathsandmarriages/deaths/datasets/carehomeresidentdeathsregisteredinenglandandwalesprovisional.

[8] Teggi D (2020). Care homes as hospices for the prevalent form of dying: an analysis of long-term care provision towards the end of life in England. Soc Sci Med.

[9] Fleming J, Calloway R, Perrels A (2017). Dying comfortably in very old age with or without dementia in different care settings - a representative “older old” population study. BMC Geriatr.

[10] NHS England (2020). Enhanced health in racfs. https://www.england.nhs.uk/publication/enhanced-health-in-care-homes-framework/.

[11] ONS (2023). RACFs and estimating the self-funding population, england - office for national statistics [internet]. https://www.ons.gov.uk/peoplepopulationandcommunity/healthandsocialcare/socialcare/articles/carehomesandestimatingtheselffundingpopulationengland/2022to2023#overview.

[12] RCGP (2016). Palliative and end of life care [internet]. https://www.rcgp.org.uk/representing-you/policy-areas/palliative-end-life-care.

[13] Waterman T (2024). Network contract directed enhanced service.

[14] GSF overview final 3 2025(1).pdf [internet]. https://www.goldstandardsframework.org.uk/cd-content/uploads/files/GSF%20Overview%20%20FINAL%203%202025%281%29.pdf.

[15] Holdsworth R, Rowley G, Farrington E (2024). Caring for dying patients in the community. BMJ.

[16] WHO (2020). Palliative care [internet]. https://www.who.int/news-room/fact-sheets/detail/palliative-care.

[17] Rhee JJ, Grant M, Senior H (2024). Facilitators and barriers to general practitioner and general practice nurse participation in end-of-life care: systematic review. BMJ Support Palliat Care.

[18] Senior H, Grant M, Rhee JJ (2024). General practice physicians’ and nurses’ self-reported multidisciplinary end-of-life care: a systematic review. BMJ Support Palliat Care.

[19] White C, Alton E (2022). The interface between primary care and care homes: General Practitioner experiences of working in care homes for older people. *Health Social Care Comm*.

[20] Chadborn NH, Devi R, Goodman C (2023). General practitioners’ role in improving health care in care homes: a realist review. Fam Pract.

[21] Banerjee A, James R, McGregor M (2018). Nursing home physicians discuss caring for elderly residents: an exploratory study. Can J Aging.

[22] NHSEngland (2023). Network contract directed enhanced service - contract specification 2023/24 – PCN requirements and entitlements.

[23] Goodman C, Froggatt K, Amador S (2015). End of life care interventions for people with dementia in care homes: addressing uncertainty within a framework for service delivery and evaluation. BMC Palliat Care.

[24] Spacey A, Scammell J, Board M (2018). End-of-life care in UK care homes: a systematic review of the literature. J Res Nurs.

[25] Groot MM, Vernooij-Dassen MJFJ, Verhagen SCA (2007). Obstacles to the delivery of primary palliative care as perceived by GPs. Palliat Med.

[26] Owen K, Hopkins T, Shortland T (2019). GP retention in the UK: a worsening crisis. Findings from a cross-sectional survey. BMJ Open.

[27] Mitchell G, Melaku M, Moss A (2022). Evaluation of a commissioned end-of-life care service in Australian aged care facilities. Prog Palliat Care.

[28] Hanratty B (2000). GP views on developments in palliative care services. Palliat Med.

[29] Bowers B, Ryan R, Kuhn I (2019). Anticipatory prescribing of injectable medications for adults at the end of life in the community: a systematic literature review and narrative synthesis. Palliat Med.

[30] Majumder M, Bowers B, Pollock K (2022). End of life care in UK RACFs - controlled drugs: systematic review and narrative synthesis. BMJ Support.

[31] Jones R, Dale J, MacArtney J (2023). Challenges experienced by GPs when providing palliative care in the UK: a systematic qualitative literature review. BJGP Open.

[32] Rosenwax L, Spilsbury K, McNamara BA (2016). A retrospective population based cohort study of access to specialist palliative care in the last year of life: who is still missing out a decade on?. BMC Palliat Care.

[33] Chapman M, Russell B, Philip J (2020). Systems of care in crisis: the changing nature of palliative care during COVID-19. *J Bioeth Inq*.

[34] Harding A, Preston N, Doherty J (2021). Developing and evaluating online COVID-centric advance care planning training and information resources for nursing staff and family members in nursing homes: the necessary discussions study protocol. BMC Geriatr.

[35] Cousins E, Preston N, Doherty J (2022). Implementing and evaluating online advance care planning training in UK nursing homes during COVID-19: findings from the Necessary Discussions multi-site case study project. BMC Geriatr.

[36] Bradshaw A, Ostler S, Goodman C (2023). Provision of palliative and end-of-life care in UK care homes during the COVID-19 pandemic: a mixed methods observational study with implications for policy. Front Public Health.

[37] Sleeman KE, Cripps RL, Murtagh FEM (2022). Change in activity of palliative care services during the Covid-19 pandemic: a multinational survey (CovPall). J Palliat Med.

[38] Etkind SN, Bone AE, Gomes B (2017). How many people will need palliative care in 2040? Past trends, future projections and implications for services. BMC Med.

[39] Teggi D (2022). End of life care in english racfs: governance, care work and the good death. PhD Thesis.

[40] Butler M, Epstein RA, Totten A (2017). AHRQ series on complex intervention systematic reviews-paper 3: adapting frameworks to develop protocols. J Clin Epidemiol.

[41] Page MJ, McKenzie JE, Bossuyt PM (2021). The PRISMA 2020 statement: an updated guideline for reporting systematic reviews. BMJ.

[42] Gough D (2007). Weight of evidence: a framework for the appraisal of the quality and relevance of evidence. Res Pap Educ.

[43] Popay J, Roberts H, Sowden A (2006). Guidance on the conduct of narrative synthesis in systematic reviews: a product from the ESRC methods programme.

[44] Pope C, Mays N (2020). Qualitative research in health care [internet]. http://ebookcentral.proquest.com/lib/cam/detail.action?docID=5987277.

[45] Thomas J, Harden A (2008). Methods for the thematic synthesis of qualitative research in systematic reviews. BMC Med Res Methodol.

[46] South J, Southby K, Freeman C (2024). Synthesising practice-based case study evidence from community interventions: development of a method. Int J Qual Methods.

[47] Handley M, Goodman C, Froggatt K (2014). Living and dying: responsibility for end-of-life care in care homes without on-site nursing provision - a prospective study. Health Soc Care Community.

[48] Dreyer A, Førde R, Nortvedt P (2011). Ethical decision-making in nursing homes: influence of organizational factors. Nurs Ethics.

[49] Kinley J, Hockley J, Stone L (2014). The provision of care for residents dying in U.K. nursing care homes. Age Ageing.

[50] Oosterveld-Vlug MG, Pasman HRW, Ten Koppel M (2019). Physician visits and recognition of residents’ terminal phase in long-term care facilities: findings from the PACE cross-sectional study in 6 EU countries. J Am Med Dir Assoc.

[51] Badger F, Plumridge G, Hewison A (2012). An evaluation of the impact of the Gold Standards Framework on collaboration in end-of-life care in nursing homes. A qualitative and quantitative evaluation. Int J Nurs Stud.

[52] Andrews N, Myall M (2023). ‘I don’t think they really link together, do they?’ An ethnography of multi-professional involvement in advance care planning in nursing homes. Age Ageing.

[53] Froggatt K, Vaughan S, Bernard C (2009). Advance care planning in care homes for older people: an English perspective. Palliat Med.

[54] Kirsebom M, Hedström M, Pöder U (2017). General practitioners’ experiences as nursing home medical consultants. Scand J Caring Sci.

[55] Tuckett A, Parker D, Clifton K (2015). What general practitioners said about the palliative care case conference in residential aged care: an Australian perspective. Part 2. Prog Palliat Care.

[56] Pulst A, Fassmer AM, Schmiemann G (2021). Unplanned hospital transfers from nursing homes: who is involved in the transfer decision? Results from the HOMERN study. Aging Clin Exp Res.

[57] Rainsford S, Johnston N, Liu W-M (2020). Palliative care Needs Rounds in rural residential aged care: a mixed-methods study exploring experiences and perceptions of staff and general practitioners. Prog Palliat Care.

[58] Sidell M, Katz JS, C K (1997). Death and dying in residential and nursing homes for older people: a multi-method approach.

[59] Vandervoort A, Houttekier D, Vander Stichele R (2014). Quality of dying in nursing home residents dying with dementia: does advanced care planning matter? A nationwide postmortem study. PLoS One.

[60] Ding J, Johnson CE, Auret K (2022). Comparison of end‐of‐life care for people living in home settings versus residential aged care facilities: a nationwide study among Australian general practitioners. Health Soc Care Community.

[61] Borbasi JAL, Tong A, Ritchie A (2021). “A good death but there was all this tension around”- perspectives of residential managers on the experience of delivering end of life care for people living with dementia. BMC Geriatr.

[62] Barańska I, Kijowska V, Engels Y (2020). Perception of the quality of communication with physicians among relatives of dying residents of long-term care facilities in 6 European countries: PACE cross-sectional study. J Am Med Dir Assoc.

[63] ten Koppel M, Pasman HRW, van der Steen JT (2019). Consensus on treatment for residents in long-term care facilities: perspectives from relatives and care staff in the PACE cross-sectional study in 6 European countries. BMC Palliat Care.

[64] Seymour JE, Kumar A, Froggatt K (2011). Do nursing homes for older people have the support they need to provide end-of-life care? A mixed methods enquiry in England. Palliat Med.

[65] Allers K, Fassmer AM, Spreckelsen O (2020). End‐of‐life care of nursing home residents: a survey among general practitioners in northwestern Germany. *Geriatrics Gerontology Int*.

[66] Balmer D, Frey R, Gott M (2020). Provision of palliative and end-of-life care in New Zealand residential aged care facilities: general practitioners’ perspectives. Aust J Prim Health.

[67] Phillips J, Davidson PM, Willcock S (2009). An insight into the delivery of a palliative approach in residential aged care: the general practitioner perspective. J Appl Gerontol.

[68] Rainsford S, Hall Dykgraaf S, Phillips C (2022). Effectiveness of telehealth palliative care Needs Rounds in rural residential aged care during the COVID-19 pandemic: a hybrid effectiveness-implementation study. Aust J Rural Health.

[69] Gorlén T, Gorlén TF, Vass M (2012). Low confidence among general practitioners in end-of-life care and subcutaneous administration of medicine. Dan Med J.

[70] Ong ACL, Sabanathan K, Potter JF (2011). High mortality of older patients admitted to hospital from care homes and insight into potential interventions to reduce hospital admissions from care homes: the Norfolk experience. Arch Gerontol Geriatr.

[71] Harasym PM, Afzaal M, Brisbin S (2021). Multi-disciplinary supportive end of life care in long-term care: an integrative approach to improving end of life. BMC Geriatr.

[72] Harasym P, Brisbin S, Afzaal M (2020). Barriers and facilitators to optimal supportive end-of-life palliative care in long-term care facilities: a qualitative descriptive study of community-based and specialist palliative care physicians’ experiences, perceptions and perspectives. BMJ Open.

[73] Grüne B, Meesters S, Bausewein C (2022). Challenges and strategies regarding sedation at the end of life in hospitals and nursing homes. J Pain Symptom Manage.

[74] Frey R, Barham S, Balmer D (2020). Palliative care delivery in residential aged care: bereaved family member experiences of the Supportive Hospice Aged Residential Exchange (SHARE) intervention. BMC Palliat Care.

[75] Bauer A-K, Fassmer AM, Zuidema SU (2024). End-of-life care in German and Dutch nursing homes: a cross-sectional study on nursing home staff’s perspective in 2022. Arch Public Health.

[76] Forbat L, Macgregor A, Spilsbury K (2024). Using palliative care needs rounds in the UK for care home staff and residents: an implementation science study. *Health Soc Care Deliv Res*.

[77] Dujardin J, Schuurmans J, Westerduin D (2021). The COVID-19 pandemic: a tipping point for advance care planning? Experiences of general practitioners. Palliat Med.

[78] Kinnock, UK Parliament (2024). Palliative care standards. https://questions-statements.parliament.uk/written-questions/detail/2024-10-11/8728.

