# Supplementary material for: General practitioner care of residential aged care facility residents at end of life: a systematic literature review and narrative synthesis
Source: BMJ Open. 2025 Nov 12;15(11):e104243. doi: 10.1136/bmjopen-2025-104243 (PMC12612765; doi:10.1136/bmjopen-2025-104243)
Supplement: online supplemental file 1 [file bmjopen-15-11-s001.docx]

**Supplementary Material 1. Full Search Strings**

**Medline**

Ovid MEDLINE[R] and Epub Ahead of Print, In-Process, In-Data-Review & Other Non-Indexed Citations, Daily and Versions[R] <1946 to February 14, 2022>

1 residential facilities/ or group homes/ or homes for the aged/ or exp nursing homes/ or institutionalization/ or Long-Term Care/ or Housing for the Elderly/ or [[[care or nursing or residential or rest or old* people* or old folk* or group or geriatric or elderly] adj2 [home or homes]] or [[long term or long-term or residential or institution*] adj care] or [[aged or elderly or geriatric or extended] adj2 care adj2 [facility or facilities]] or [[aged or elderly] adj3 [home or homes]]].mp.

2 exp Terminal Care/ or exp Palliative Care/ or exp "Hospice and Palliative Care Nursing"/ or exp death/ or exp Palliative Medicine/ or exp Terminally Ill/ or [[end adj2 life] or [[final* or last*] adj1 [hour* or day* or minute* or week* or month* or moment*]] or palliat* or terminal* or [end adj stage] or dying or [body adj2 [shutdown or shut* down or deteriorat*]] or deathbed].mp. 3 exp General Practitioners/ or exp physicians, family/ or [gp or general practi* or [[family or primary care or primary healthcare] adj3 [doctor* or physician*]]].mp.

4 1 and 2 and 3

# **Embase**

## 1 exp *long term care/ or exp *residential care/ or exp *nursing home/ or exp *Homes for the aged/ or [[[care or nursing or residential or rest or old* people* or old folk* or group or geriatric or elderly] adj2 [home or homes]] or [[long term or long-term or residential or institution*] adj care] or [[aged or elderly or geriatric or extended] adj2 care adj2 [facility or facilities]] or [[aged or elderly] adj3 [home or homes]]].ti,ab

## 2 exp *Terminal Care/ or exp *Palliative therapy/ or exp *palliative nursing/ or exp *palliative treatment/ or exp *hospice care/ or exp *hospice/ or exp *Terminally Ill Patient/ or exp *dying/ or [[end adj2 life] or [[final* or last*] adj1 [hour* or day* or minute* or week* or month* or moment*]] or palliat* or terminal* or [end adj stage] or dying or [body adj2 [shutdown or shut* down or deteriorat*]] or deathbed].ti,ab.

## 3 [gp or general practi* or [[family or primary care or primary healthcare] adj3 [doctor* or physician*]]].ti,ab. or exp *general practitioner/

## 4 1 and 2 and 3

## **CINAHL via Ebsco**

| S4 | S1 AND S2 AND S3 |
| --- | --- |
| S3 | [[end N2 life] or [[final* or last*] N1 [hour* or day* or minute* or week* or month* or moment*]] or palliat* or terminal* or [“end stage”] or dying or [body N2 [shutdown or shut* down or deteriorat*]] or deathbed] or [MH "Terminal Care+"] OR [MH "Palliative Care"] OR [MH "Hospice and Palliative Nursing"] OR [MH "Hospice Patients"] OR [MH "Hospices"] OR [MH "Hospice Care"] OR [MH "Terminally Ill Patients"] OR [MH "Death+"] |
| S2 | [[[care or nursing or residential or rest or old* people* or old folk* or group or geriatric or elderly] n2 [home or homes]] or [[long term or long-term or residential or institution*] n1 care] or [[aged or elderly or geriatric or extended] n2 care n2 [facility or facilities]] or [[aged or elderly] n3 [home or homes]]] or [MH "Nursing Home Patients"] OR [MH "Nursing Homes+"] OR [MH "Long Term Care"] |
| S1 | [gp or "general practition*" or [family adj [doctor* or physician*]] or [[primary care or primary healthcare] n5 doctor*]] or [MH "Physicians, Family"] |

Bottom of Form

# **PsycINFO via Ebsco**

| S4 | S1 AND S2 AND S3 |
| --- | --- |
| S3 | TI [gp or general practi* or [[family or primary care or primary healthcare] n3 [doctor* or physician*]]] or AB [gp or general practi* or [[family or primary care or primary healthcare] n3 [doctor* or physician*]]] or [DE "General Practitioners"] OR [DE "Family Physicians"] |
| S2 | TI [ [[care or nursing or residential or rest or old* people* or old folk* or group or geriatric or elderly] n2 [home or homes]] or [[long term or long-term or residential or institution*] n1 care] or [[aged or elderly or geriatric or extended] n2 care n2 [facility or facilities]] or [[aged or elderly] n3 [home or homes]] ] or AB [ [[care or nursing or residential or rest or old* people* or old folk* or group or geriatric or elderly] n2 [home or homes]] or [[long term or long-term or residential or institution*] n1 care] or [[aged or elderly or geriatric or extended] n2 care n2 [facility or facilities]] or [[aged or elderly] n3 [home or homes]] ] OR [DE "Nursing Home Residents" OR DE "Nursing Homes" OR DE "Nursing Home Residents"] OR [DE "Residential Care Institutions" OR DE "Halfway Houses" OR DE "Hospitals" OR DE "Nursing Homes" OR DE "Orphanages"] |
| S1 | TI [[end N2 life] or [[final* or last*] N1 [hour* or day* or minute* or week* or month* or moment*]] or palliat* or terminal* or [“end stage”] or dying or [body N2 [shutdown or shut* down or deteriorat*]] or deathbed] or AB [[end N2 life] or [[final* or last*] N1 [hour* or day* or minute* or week* or month* or moment*]] or palliat* or terminal* or [“end stage”] or dying or [body N2 [shutdown or shut* down or deteriorat*]] or deathbed] or [[DE "Terminally Ill Patients"] OR [DE "Palliative Care"]] OR [DE "Death and Dying" OR DE "Euthanasia" OR DE "Parental Death"] OR [DE "Hospice"] |

**Bottom of Form**

# **Web of Science Core Collection**

TS=[gp or general practi* or [[family or primary care or primary healthcare] near3 physician*] or [[family or primary care or primary healthcare] near3 doctor* ] ]

AND

TS=[[end near2 life] or [[final* or last*] near1 [hour* or day* or minute* or week* or month* or moment*]] or palliat* or terminal* or [“end stage”] or dying or deathbed or [body near2 deteriorat*] or [body near2 "shut* down"] or [body near2 shutdown]]

AND

TS=[ [[care or nursing or residential or rest or old* people* or "old folk*" or group or geriatric or elderly] and homes] or [[care or nursing or residential or rest or old* people* or "old folk*" or group or geriatric or elderly] and home]or [["long term" or long-term or residential or institution*] and care] or [[aged or elderly or geriatric or extended] and "care facility"] or [[aged or elderly or geriatric or extended] and "care facilities"] ]


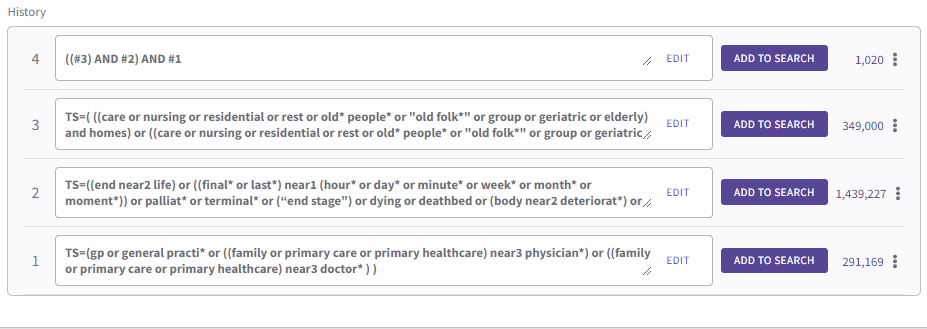


# **Scopus**

title-abs-key [ [[care or nursing or residential or rest or “old* people*” or “old folk*” or group or geriatric or elderly] w/2 [home or homes]] or [[“long term” or long-term or residential or institution*] w/1 care] or [[aged or elderly or geriatric or extended] w/2 care w/2 [facility or facilities]] or [[aged or elderly] w/3 [home or homes]] ] and title-abs-key [gp or “general practi*” or [[family or “primary care” or “primary healthcare”] w/3 [doctor* or physician*]]] and title-abs-key[[end w/2 life] or [[final* or last*] w/1 [hour* or day* or minute* or week* or month* or moment*]] or palliat* or terminal* or ["end stage"] or dying or deathbed or [body w/2 deteriorat*] or [body w/2 "shut* down"] or [body w/2 shutdown]]

# **NHS Evidence**

|  | *March 2021* | *Feb 2022* |
| --- | --- | --- |
| *"nursing home" "end of life" "general practitioner"* | *91* | *5* |
| "homes for the aged" "end of life" "general practitioner" | 1 | 0 |
| "homes for the aged" "end of life" "family doctor" | 0 | 0 |
| "nursing home" "end of life" "family doctor" | 16 | 0 |
| "old folks home" "end of life" "family doctor" | 0 | 0 |
| "old folks home" "end of life" "general practitioner" | 0 | 0 |
