# Supplementary material for: General practitioner care of residential aged care facility residents at end of life: a systematic literature review and narrative synthesis
Source: BMJ Open. 2025 Nov 12;15(11):e104243. doi: 10.1136/bmjopen-2025-104243 (PMC12612765; doi:10.1136/bmjopen-2025-104243)
Supplement: online supplemental file 2 [file bmjopen-15-11-s002.docx]

**Supplementary Material 2. Data Extraction Tool**

| **Data Extraction Tool**  **General Practitioner Care of Care Home Residents Approaching End of Life: a mixed-methods narrative synthesis of the evidence** | |
| --- | --- |
| **Details of publication** | |
| First author |  |
| Date |  |
| Reference |  |
| Type of Literature (peer reviewed/grey) |  |
| **Introduction** | |
| Aims |  |
| **Study participants** | |
| Country of study |  |
| Type of study |  |
| Recruitment |  |
| - Setting |  |
| - Characteristics of participants:   - Type (GP, MDT, PCN, care home staff, residents, family/carers, other)   - Number   - Demographics |  |
| **Methods** | |
| - Date of fieldwork |  |
| - Research methods |  |
| - Analysis |  |
| **Key findings relevant to review** | |
| a) In what ways do GPs provide care? |  |
| c) What is the quality of GP care?  i) overall assessment of quality of GP care |  |
| ii) what do GPs do well? | . |
| iii) what do GPs do less well? |  |
| d) What initiatives have sought to optimise GP care? |  |
| e) What works in improving GP care? |  |
| f) How has GP care changed during / following the COVID-19 pandemic? |  |
| g) Participants’ suggestions to improve GP care |  |
| **Author(s) conclusion(s)** |  |
| **Comments** |  |
| **Reviewers’ quality assessment of research (Gough WoE, 2007)** | |
| **Weight of Evidence A**  Coherence and integrity of the evidence *in its own terms* |  |
| **Weight of Evidence B**  Appropriateness *of form of evidence* for answering review question |  |
| **Weight of Evidence C**  *Relevance of the evidence* for answering review question |  |
| **Weight of Evidence D**  *Overall assessment* of study contribution to answering review question |  |
