# Supplementary material for: General practitioner care of residential aged care facility residents at end of life: a systematic literature review and narrative synthesis
Source: BMJ Open. 2025 Nov 12;15(11):e104243. doi: 10.1136/bmjopen-2025-104243 (PMC12612765; doi:10.1136/bmjopen-2025-104243)
Supplement: online supplemental file 3 [file bmjopen-15-11-s003.docx]

**Supplementary Material 3: Table Showing the Methodological Detail and Weight of Evidence for the Included Papers**

|  |  |  |  |  |  |  |  |  |
| --- | --- | --- | --- | --- | --- | --- | --- | --- |
| **First Author** | **Date** | **Focus** | **Country** | **Method** | **Sample** | **Sample Size** | **SR Questions Answered** | **Overall WoE** |
| 1. Allers | 2020 | Palliative & End of Life Care (PEoLC), Residential Aged Care (RAC) | Germany | Quantitative Survey | GPs | N=375 | 2,3,4 | H-H |
| 2.Andrews | 2023 | ACP in RAC | UK | Mixed, Observation, Interviews, Document Review | Residents, Relatives, Care Home Staff (CHS), Health/Social Care | (n = 6), (n = 4), (n = 19), (n = 7) | 2,3,4 | H-H |
| 3. Badger | 2012 | Gold Standards | UK | Mixed, Quantitative survey, Qualitative interviews/focus groups | CH Managers | Quantitative N=49 pre/post surveys, Qualitative N=75 | 1,2,3,4 | H-H |
| 4. Balmer | 2020 | PEoLC provision | New Zealand | Qualitative Interviews | GPs | N=17 | 2,4 | H-H |
| 5. Banerjee | 2018 | Caring for RAC | Canada, Germany, Norway, Sweden, UK, US | Qualitative Ethnography | GPs | N=18 | 1,3,4 | H-H |
| 6. Baranska | 2020 | Communication | PACE: Belgium, Italy, Netherlands, UK, Finland | Quantitative Survey | Relatives | N=761 | 1,2 | H-H |
| 7. Bauer | 2024 | PEoLC RAC | Germany, Netherlands | Quantitative Survey | Nursing Staff Managers | N=301 | 1,2 | M-M |
| 8. Borbasi | 2021 | Care Home Managers perception of PEoLC | Australia | Qualitative Interviews | CH Managers | Focus groups N=16,  Interviews N=4 | 1,3,4 | H-H |
| 9. Ding | 2022 | Compare home care with RAC | Australia | Quantitative Survey | GPs | N=62 | 1,4 | M-M |
| 10. Dreyer | 2011 | Decision Making | Norway | Qualitative Interviews | Nurses/Physicians | N=10 Nurses,  N=9 GPs | 1,2,3,4 | H-H |
| 11. Dujardin | 2021 | Advance Care Planning | Netherlands | Qualitative Interviews | GPs | N=15 | 3,5 | H-H |
|  |  |  |  |  |  |  |  |  |
| 12. Forbat | 2024 | Palliative Care Needs Rounds Implementation | UK | Qualitative Interviews, Surveys | CH Staff, Specialist PC Staff, Public/Patient Group/Relatives | N=58 Interviews with CH/SPC, PPI survey N=11, N=13 relative surveys, N=171/81 CH staff survey | 2,3,4 | H-H |
| 13. Frey | 2020 | PEoLC RAC | New Zealand | Qualitative Interviews | Relatives | N=18 | 4 | H-H |
| 14. Froggatt | 2009 | ACP in RAC | PACE: Belgium, Italy, Netherlands, UK, Finland | Mixed Survey/Interviews | CH Managers | N=213 Survey N=15 Interviews | 1,4, | M-M |
| 15. Gorlen | 2013 | Death in RAC | Denmark | Qualitative Interviews | Nurses/Assistants | N=2 Nurses, N=14 Nurse Assistants | 3,4 | H-H |
| 16. Grune | 2021 | Sedation in RAC | Germany | Qualitative Interviews | GPs, CH Nurses  Hospital Dr, Hospital Nurses | N=12, N=12, N=12, N=13 | 3,4 | H-H |
| 17. Handley | 2014 | PEoLC in RAC | UK | Mixed Note search/Interviews | GPs, Community Specialist PC, Nurses, CHStaff, Residents | N=121 resident experiences tracked, Interviews N=63, N=CHS, N=19 NHS Staff | 1,3,4 | H-H |
| 18. Harasym | 2020 | Barriers and facilitators | Canada | Qualitative Interviews | GPs, Specialist PC Dr | N=18 Community Physicians, N=5 Specialist PC Physicians | 3,4 | H-H |
| 19. Harasym | 2021 | Multidisciplinary Approach | Canada | Delphi | GPs, multidisciplinary | N=61 Round 1, N=35 Round 2 | 3,4 | M-M |
| 12. Kinley | 2014 | PEoLC RAC | UK | Quantitative Note Searching | Decendens | N=2444 | 2, | L-L |
| 21. Kirsebom | 2017 | GP Experience | Sweden | Qualitative Interviews | GPs | N=15 | 1,3,4 | H-H |
| 22. Mitchell | 2022 | Evaluation | Australia | Mixed, Chart Audits/Focus groups | Deaths/CHStaff/Family/GPs | N=68 Deaths, Ns for Interviews/Focus Groups not reported | 5 | H-H |
| 23. Nilsen | 2024 | CH Resident Care during COVID-19 | Norway | Qualitative Interviews | Physicians, Nurses, Relatives | N=35 | 5 | H-H |
| 24. Ong | 2011 | Reducing Hospital Admissions | UK | Mixed, Note search/Interviews | CH Staff |  | 2,3,4, | M-M |
| 25. Oosterveld-Vlug | 2019 | GP recognition last phase of life | PACE: Belgium, Italy, Netherlands, UK, Finland | Quantitative survey | GP, CH Manager | N=505 | 1,2 | H-H |
| 26. Phillips | 2009 | GP Perspective of an Evaluation | Australia | Qualitative Focus Groups | GPs | N=13 | 2,3,4 | M-M |
| 27. Pulst | 2021 | Unplanned Hospitalisations | Germany | Quantitative survey | CH Staff | N=534 | 1, | M-M |
| 28. Rainsford | 2020 | Specialist PEoLC Needs Rounds | Australia | Mixed, Survey and Interviews | CH Staff/GPs | N=61 surveys, N=8 CHS, N=3 GPs | 1,3,4 | H-H |
| 29.  Rainsford | 2022 | Specialist PEoLC Needs Rounds | Australia | Interviews | CH Managers/Registered Nurses/ Care Assistant | N=2  N=4  N=1 | 3,4,5 | M-M |
| 30. Sidell | 1997 | Death & Dying in RAC | UK | Mixed, Survey, Interviews, Case Studies | CH Staff, GPs | N=1000 Surveys, N=100 Interviews, N=12 Case Studies | 1,3,4 | H-H |
| 31. Seymour | 2011 | Can RAC provide PEoLC | UK | Mixed, Survey & case studies | CH Staff, Community Practitioners | N=82 Questionnaires, N=2 Case Studies | 2,3,4 | H-H |
| 32. Ten Koppel | 2019 | Consensus of Treatment | Australia | Qualitative Interviews | Relatives, Care Home Staff | N=840 Relatives, N=1384 CHS | 2 | M-M |
| 33. Tuckett | 2013 | Palliative care case conference | Australia | Qualitative Interviews | GPs | N=11 | 1,4, | H-H |
| 34.  Tuckett | 2015 | Palliative care case conference | Australia | Qualitative Interviews | GPs | N=11 | 3,4 | M-M |
| 35. Vandervoort | 2014 | Quality of dying | Belgium | Quantitative survey | GPs, nurses, relatives, CHM | N=101 Nurse, N=101 CHM | 1,2 | M-M |
