# Supplementary figures and images for: General practitioner care of residential aged care facility residents at end of life: a systematic literature review and narrative synthesis
Source: BMJ Open. 2025 Nov 12;15(11):e104243. doi: 10.1136/bmjopen-2025-104243 (PMC12612765; doi:10.1136/bmjopen-2025-104243)

Supplementary Material 4: Included Papers by Semantic Themes


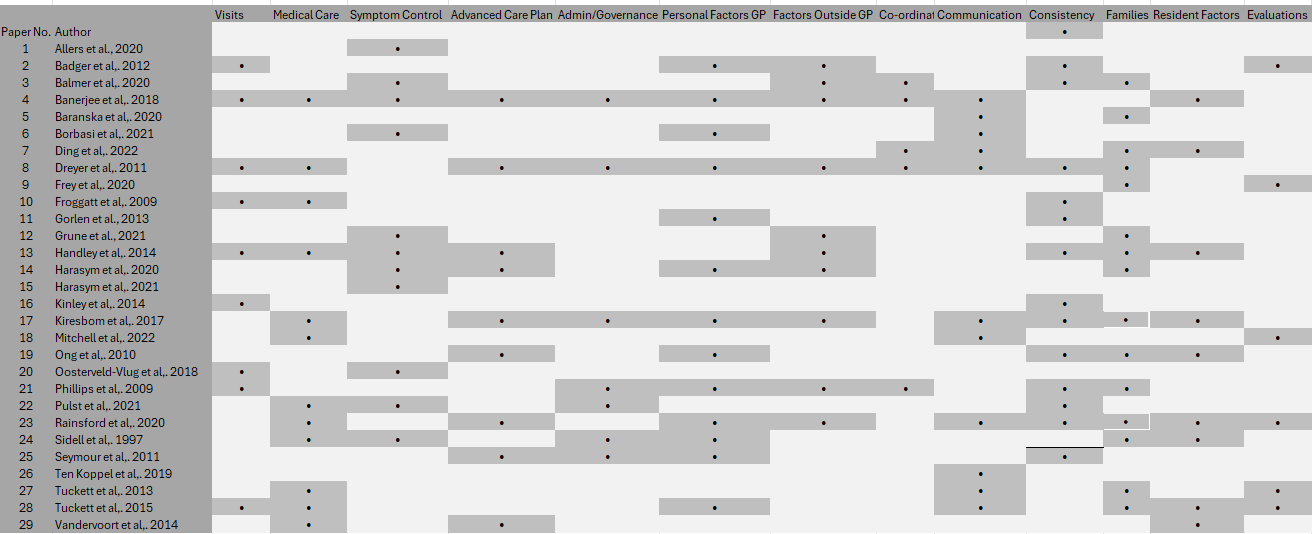

Supplement: online supplemental file 4 [file bmjopen-15-11-s004.docx]
