# Supplementary material for: General practitioner care of residential aged care facility residents at end of life: a systematic literature review and narrative synthesis
Source: BMJ Open. 2025 Nov 12;15(11):e104243. doi: 10.1136/bmjopen-2025-104243 (PMC12612765; doi:10.1136/bmjopen-2025-104243)
Supplement: Supplementary file 5 [file bmjopen-2025-104243.reviewer_comments.pdf]

PEER REVIEW HISTORY

BMJ Open publishes all reviews undertaken for accepted manuscripts. Reviewers are asked to complete a checklist review form and are provided with free text boxes to elaborate on their assessment. These free text comments are reproduced below.

ARTICLE DETAILS

Title (Provisional)

General Practitioner care of Residential Aged Care Facility residents at End of Life: a systematic literature review and narrative synthesis

Authors

Browne, Susannah; Kelly, Michael P; Bowers, Ben; Kuhn, Isla; Duschinsky, Robbie; Daniels, Charles; Barclay, Stephen

---

VERSION 1 - REVIEW

**Reviewer**	**1**
**Name**	**Laranjeira, Carlos**
**Affiliation**	**Polytechnic Institute of Leiria, School of Health Sciences**
**Date**	**06-Jul-2025**
**COI**	**None**

I appreciate the opportunity to review this manuscript and hope my comments assist in the revision process. The material is interesting, and the topic is timely and relevant. The method seems to have been followed, and the authors were well-positioned to conduct the analysis. However, some areas need clarification:

- The abstract should be informative, the background does not explain the question of this review and the answer which the authors search for it. Please provide more information about the methods in the abstract.

- Keywords: are these keywords Mesh terms?

- First, I suggest dividing the section "INTRODUCTION" into three components, respectively introduction (explain the general argument of the paper, without going into specific details) background (situate the study concepts within the context of extant knowledge, discuss the international relevance of the concepts) and purpose, creating greater clarity in the analysis of the reader. What knowledge does this paper add from an international perspective? How this/your study is increasing our understanding/knowledge?

- Describe more deeply the rationale for the review in the context of what is already known.

Method

- Do you use any software to manage the flow of citations and articles through the review?

- Describe more precisely the process for coding data (e.g. line by line coding to search for concepts).

- Describe how comparisons were made within and across studies (e.g. subsequent studies were coded into pre-existing concepts, and new concepts were created when deemed necessary).

- Explain whether the process of deriving the themes or constructs was inductive, deductive or abductive. The authors stated that "inductive narrative synthesis" was conducted; however, data presentation seems more like a hybrid approach (deductive and inductive).

Findings

Present rich, compelling and useful results that go beyond a summary of the primary studies (e.g. new interpretation, models of evidence, conceptual models, analytical framework, development of a new theory or construct).

Discussion

- I believe there should be better integration of the results with the existing literature.

- The recommendations/implications for practice/research/education should have been approached in greater depth. I believe this will considerably strengthen this paper.

- Theoretical and methodological limitations should be emphasised more deeply.

- Give the reader some deeper insight in your conclusion section (rename summary to conclusion).

CHECKLIST FOR STYLE

The manuscript will serve a broad audience of students, researchers, and practitioners; however, the manuscript needs to be carefully and attentively proofread. The English of this manuscript should be reviewed by a native-English speaker.

- Overall, I strongly recommend double-checking PRISMA checklist and ensuring that every single item is included in your paper (if not, explain).

**Reviewer**	**2**
**Name**	**Green, Richard**
**Affiliation**	**University of Surrey, School of Health Sciences**
**Date**	**20-Aug-2025**
**COI**	**None**

Dear authors,

Thank you for the opportunity to read your manuscript. I found it to be well-written and interesting.

Below are some comments. I do not personally consider any of these to require significant revisions, and some of the more substantive points are written as suggestions for your consideration for how you may wish (or not wish) to address them, or as you may be guided by the journal editor.

Abstract

Conclusion section- with enablers and challenges (missing word: at?) all levels within existing care systems.

Introduction is clear and well presented, providing justification for the systematic review.

Methods

Information Sources

"'Care Home' was defined as any residential setting with or

without onsite nursing provision, which is not a resident's own home." - Perhaps a small thing, but some may consider a care home to be their "own home"- perhaps replace with something like "own domestic dwelling".

Study selection and data extraction

Who undertook the title screening? All authors?

Also, I suggest that if abstract screening was done on 16 papers then perhaps also clarify what percentage this is of the total number of abstracts that were screened- (106 I think?) as this might be confused with the total number of overall papers screened (title and abstract).

Quality Appraisal

What were the scores for the WoE appraisal- they don't appear to be signposted to in appendices? NOTE: I see you describe this further below in the manuscript, perhaps signpost at this point for clarity for the reader (or here and further below).

Data Synthesis

How is the analysis inductive if the analysis is being structured by your data extraction table and then stratified by the review questions? Is this not some kind of framework for analysis?

Patient and public involvement

Can you give any brief description of the impact that these advisors and/or activities had in shaping this work? Otherwise this reads more like a tickbox exercise.

"The review was registered with PROSPERO [CRD42021254141]" - I think you are repeating yourself here, already described further above.

RESULTS

"Due to the small sample sizes and heterogeneity of methods and participants across the studies a descriptive approach to reporting was adopted to avoid bias." - not sure what you mean here?

Table 4 - In the Key, should the questions be phrased the same as they are further above when first introduced?

Table 5 - this might work better in landscape format and I'm not sure it is adding much in the main text. Perhaps make an appendix item and just comment on any interesting trends in the table and then cite the supplementary file number.

Overall this is a detailed and comprehensive account of different enabling and challenging factors, as well as addressing other research questions posed.

DISCUSSION

Given that you have completed a quality appraisal exercise, is there anything that can be said about which of these factors has stronger weighting (or evidence backing) than others?

There is such a volume of factors reported in the Results, that it would perhaps be beneficial to signpost the reader to those that are most commonly (and/or strongly) evidenced from the included papers, as per the quote below:

"GPs are at the centre of PEOLC for residents; we highlight the multifactorial dependencies that intervene in its practice."

...

Page 20 of 43, line 1 - here you outline the 'main factors', but how are these determined?

Discussion of what quality is in GP PEOLC and what inferences we might draw is good to read. Also discussion of gaps in the literature.

VERSION 1 - AUTHOR RESPONSE

	Comments	Susannah's Response	Page of Track Changes version of paper
**Abstract**			
Reviewer 1	The abstract should be informative. The background does not explain the question of this review and the answer which the authors search for it	Thank you. I have rephrased the objectives to say: "To review the published evidence concerning how GPs provide this care: what they do and the quality, challenges and facilitators of that care" to make the aims clearer.	2
	Please provide more information about the methods in the abstract.	Thank you. Design now states: Systematic Review and narrative synthesis using the	2

		Preferred Reporting Items for Systematic Reviews and Meta-Analysis (PRISMA)	
Reviewer 2	Conclusion section- with enablers and challenges (missing word: at?) all levels within existing care systems.	I have added "at" all levels within…	3
Keywords			
Reviewer 1	Keywords: are these keywords Mesh terms?	Thank you. I have amended to Keywords (MESH) to make this clear. We have changed the title and text to use the MESH term Residential Aged Care Facility replacing the previous term Care Home.	3
**Introduction**			
Reviewer 1	First, I suggest dividing the section "INTRODUCTION" into three components, respectively: **Introduction** explain the general argument of the paper, without going into specific details; **Background** situate the study concepts within the context of extant knowledge; **Purpose**, creating greater clarity in the analysis of the reader. What knowledge does this paper add from an international perspective. How is this study increasing understanding/knowledge?	We have discussed this suggestion from Reviewer 1. In the light of the comment of Reviewer 2 that the Introduction is clear and well presented, we have not changed the structure as suggested by Reviewer 1. We have however sought to address the comments of Reviewer 1, particularly concerning the extant literature	3&4
Reviewer 2	Introduction is clear and well presented, providing justification for the systematic review.	Thank you. As mentioned above, we have left the structure and content of the Introduction largely unchanged	
**Method**			
Reviewer 1	Do you use any software to manage the flow of citations and articles through the review?	Yes, we used Endnote: I have added this to the paper	8
	Describe more precisely the process for coding data (e.g. line by line coding to search for concepts).	Thank you. Line by line coding was used to sort the data into semantic themes. These were then conceptualised into macro, meso, micro levels of the system of care to aid reporting. I have added this to the paper	9

	Describe how comparisons were made within and across studies (e.g. subsequent studies were coded into pre-existing concepts, and new concepts were created when deemed necessary).	Thank you. Following coding the process of sorting the data into themes as described above followed a process of coded text being sorted in pre-existing themes, with new concepts and themes added when deemed necessary. I have amended the text to explain this process	9
	Explain whether the process of deriving the themes or constructs was inductive, deductive or abductive. The authors stated that "inductive narrative synthesis" was conducted; however, data presentation seems more like a hybrid approach (deductive and inductive).	Thank you for this helpful comment. The process was indeed a hybrid one, in part deductive based on the review questions while also being inductive to allow new themes to emerge. The text has been amended accordingly	9
Reviewer 2	"‘Care Home’ was defined as any residential setting with or	I have amended care home and nursing home references throughout the text (and the title of the paper) to Residential Aged Care Facility which is a MeSH term	1 and throughout
	Who undertook the title screening? All authors?	SBr screened the titles. I have amended text to be clearer	
	Also, I suggest that if abstract screening was done on 16 papers then perhaps also clarify what percentage this is of the total number of abstracts that were screened- (106 I think?) as this might be confused with the total number of overall papers screened (title and abstract)	We have amended the text to make it clear that 62% of abstracts were double screened by SBr and BB	8
	What were the scores for the WoE appraisal- they don't appear to be signposted to in appendices? NOTE: I see you describe this further below in the manuscript, perhaps signpost at this point for clarity for the reader (or here and further below).	Thank you. I have amended the text to tell reader were WoE scores can be found	9
	How is the analysis inductive if the analysis is being structured by your data extraction table and then stratified by the review questions? Is this not some kind of framework for analysis?	Thank you. As indicated above, the analysis was hybrid. It was initially deductive in that the data was initially sorted into the review questions addressed. It was also inductive in that the semantic themes were then identified and then the conceptual themes were developed from that. We have amended the text to make this clearer	9

	Can you give any brief description of the impact that these advisors and/or activities had in shaping this work? Otherwise this reads more like a tickbox exercise.	Thank you. We have made their contribution clearer in the revised text	9
	"The review was registered with PROSPERO [CRD42021254141]" - I think you are repeating yourself here, already described further above.	Thank you. This repetition has been removed from the revised text	9
**Results**	without onsite nursing provision, which is not a resident's own home." - Perhaps a small thing, but some may consider a care home to be their "own home"- perhaps replace with something like "own domestic dwelling".	Thank you. We have made this change in the revised text	7
Reviewer 1	Present rich, compelling and useful results that go beyond a summary of the primary studies (e.g. new interpretation, models of evidence, conceptual models, analytical framework, development of a new theory or construct).	Thank you. We are pleased to see that our results are seen to be rich and useful and go beyond a simple summary of the primary studies	
	The recommendations/implications for practice/research/education should have been approached in greater depth. I believe this will considerably strengthen this paper.	Thank you. We have developed this section further	20
	Theoretical and methodological limitations should be emphasised more deeply.	Thank you. We have described the limitations of the current literature and the potential limitations of our approach in the revised text	3
Reviewer 2	"Due to the small sample sizes and heterogeneity of methods and participants across the studies a descriptive approach to reporting was adopted to avoid bias." - not sure what you mean here?	Thank you. We have amended the text to make this clearer. Due to the methodological limitations and small sample sizes of the included studies we adopted a descriptive approach to reporting the studies. It was not possible to undertake meta-analysis or meta-synthesis	9

	Table 4 - In the Key, should the questions be phrased the same as they are further above when first introduced?	Thank you. We have amended the text	11
	Table 5 - this might work better in landscape format and I'm not sure it is adding much in the main text. Perhaps make an appendix item and just comment on any interesting trends in the table and then cite the supplementary file number.	Thank you. We agree and have taken this out from the main paper and referred to it as supplementary material 4	SM 4
	Overall this is a detailed and comprehensive account of different enabling and challenging factors, as well as addressing other research questions posed.	Thank you for this affirming comment. This was our aim in undertaking the review	
**Discussion**			
Reviewer 2	Given that you have completed a quality appraisal exercise, is there anything that can be said about which of these factors has stronger weighting (or evidence backing) than others?	Thank you. We have amended the text to say that the themes with the greatest Weight of Evidence were "consistency", "communication", "working with families" and "symptom control".	11
	There is such a volume of factors reported in the Results, that it would perhaps be beneficial to signpost the reader to those that are most commonly (and/or strongly) evidenced from the included papers, as per the quote below:	Thank you for this very helpful suggestion. I have amended the text to signpost and highlight the five most salient themes that either facilitate or challenge care provision.	11
	"GPs are at the centre of PEOLC for residents; we highlight the multifactorial dependencies that intervene in its practice	Thank you: we have amended the text as above	11
	Page 20 of 43, line 1 - here you outline the 'main factors', but how are these determined?	Thank you. I have removed the word 'main' to indicate the diversity of factors involved	18
	Discussion of what quality is in GP PEOLC and what inferences we might draw is good to read. Also discussion of gaps in the literature.	Thank you for this affirming comment	

Conclusion

Reviewer 1	Give the reader some deeper insight in your conclusion section (rename summary to conclusion).	Thank you. We have developed the summary section further. Happy to change this to "Conclusion" if that fits with the journal house-style

Style

Reviewer 1	the manuscript needs to be carefully and attentively proofread	We have proof-read the amended text with care
	The English of this manuscript should be reviewed by a native-English speaker.	We have reviewed the text carefully: all the authors are native English speakers
	I strongly recommend double-checking PRISMA checklist and ensuring that every single item is included in your paper (if not, explain).	We have checked the PRISMA checklist and confirm that all items are included
Reviewer 2	I found it to be well-written and interesting.	Thank you for this affirming comment

VERSION 2 - REVIEW

**Reviewer**	**1**
**Name**	**Laranjeira, Carlos**
**Affiliation**	**Polytechnic Institute of Leiria, School of Health Sciences**
**Date**	**11-Oct-2025**
**COI**	

I believe that the review conducted has greatly enhanced the quality of the study. Additionally, I do think that the author(s) appropriately address the broad questions that were asked.
